# Dietary protein and plasma total homocysteine, cysteine concentrations in coronary angiographic subjects

**DOI:** 10.1186/1475-2891-12-144

**Published:** 2013-11-07

**Authors:** Yunjun Xiao, Yuan Zhang, Min Wang, Xinrui Li, Min Xia, Wenhua Ling

**Affiliations:** 1 Guangdong Provincial Key Laboratory of Food, Nutrition and Health, Department of Nutrition, School of Public Health, Sun Yat-sen University, Number 74 Zhongshan Road 2, 510080 Guangzhou, Guangdong, China; 2 Department of Nutrition and Food Hygiene, Shenzhen Center for Disease Control and Prevention, Shenzhen, Guangdong, China; 3 Department of Cardiology, Guangzhou Military General Hospital, Guangzhou, Guangdong, China

**Keywords:** Diet, Dietary protein, Total homocysteine, Total cysteine, Coronary artery disease

## Abstract

**Background:**

Dietary patterns are associated with plasma total homocysteine (tHcy) concentrations in healthy populations, but the associations between dietary protein and tHcy, total cysteine (tCys) in high risk populations are unclear. We therefore examined the association between dietary protein and tHcy and tCys concentrations in coronary angiographic subjects.

**Methods:**

We conducted a cross-sectional study of 1015 Chinese patients who underwent coronary angiography (40–85 y old). With the use of food-frequency questionnaires, we divided the total protein intakes into high animal-protein and high plant-protein diets. Circulating concentrations of tHcy and tCys were simultaneously measured by high-performance liquid chromatography with fluorescence detection.

**Results:**

We found that high animal-protein diet was positively associated with hyperhomocysteinemia after adjustment for potential confounders, with the subjects in the highest quartile of intake having the greatest increase in risk (OR: 4.14, 95% CI: 2.67-6.43), whereas high plant-protein diet was inversely related to hyperhomocysteinemia, with a higher intake being protective. Compared with the first quartile of intake, the adjusted OR was 0.59 (95% CI: 0.38-0.91) for the fourth quartile. The total protein intake was positively associated with the risk of hypercysteinemia and the participants in highest quartile had significant OR of 1.69 (95% CI: 1.02-2.87) compared with those in lowest quartile. In multivariate linear regression analyses, high animal-protein and total-protein intakes were positively associated with plasma tHcy and tCys concentrations. The plant-protein intake was a negative determinant of plasma tHcy concentrations.

**Conclusions:**

High animal-protein diet was positively associated with high tHcy concentrations, whereas high plant-protein diet was inversely associated with tHcy concentrations. Furthermore the total protein intake was strongly related to tCys concentrations.

## Background

Plasma total homocysteine (tHcy) have been considered as a risk factor for cardiovascular disease (CVD) [1]. However, most recent large-scale clinical trials have demonstrated that the therapeutic use of folate and B vitamins aimed at reducing tHcy levels does not reduce the risk of CVD [2]. Furthermore, another amino acid containing a sulf-group cysteine, structurally like homocysteine, was reported to be a risk factor of CVD [3-5], but in a prospective study, plasma total cysteine (tCys) was not an independent risk factor of CVD [6]. Homocysteine can be remethylated to methionine by methionine synthase in a reaction that requires methyltetrahydrofolate as a methyl donor and vitamin B_12_ as an enzyme cofactor. Alternatively, homocysteine can be transsulfurated to cysteine in reactions that require vitamin B_6_[7]. The major causes of hyperhomocysteinemia include impairment of renal function, deficiencies of plasma folate, vitamin B_12_, and vitamin B_6_, and genetic factors, such as a genetic mutation (nucleotide C677T, alanine to valine substitution) in the enzyme methylenetetrahydrofolate reductase (MTHFR) [8,9]. Furthermore, certain lifestyles and dietary factors have been identified as predictors of tHcy concentrations in healthy subjects [10,11]. Smoking and alcohol consumption have been associated with increasing tHcy concentrations [12,13], whereas dietary and plasma folate have been associated with lower tHcy concentrations [14]. Recently it has been reported that Western diet patterns (high-energy, high-fat, and high-protein diet) are strongly associated with increased risk of CVD [15,16]. On the other hand, the prudent pattern contained high amounts of fruits, vegetables, legumes, fish, poultry, and low fat diary products and showed protective effects for chronic diseases. However, whether dietary proteins influence plasma tHcy and tCys concentrations in CVD are unclear.

Industrialization and urbanization have led to the transition from plant-based dietary patterns to animal-based dietary patterns that may lead to the development of chronic diseases in developing countries like China. Therefore, understanding patterns of dietary intake and their association with the risk factors for CVD would be helpful in developing prevention programs for overcoming such health issues. Although a numbers of studies have been carried out in developed countries to determine the association between tHcy and dietary patterns [13,14,17], there are very few studies from developing countries reporting the relationship of dietary proteins with plasma tHcy and tCys concentration in coronary artery disease (CAD) patients in China. The purpose of this study was to investigate the relationship of dietary protein intake with plasma tHcy and tCys concentrations in CAD patients.

## Methods

### Study population

The current study was conducted in a nested fashion within the ongoing Guangzhou cardiovascular disease study [18]. The study was a collaboration of the Guangzhou Military General Hospital, Zhujiang Hospital, Sun Yat-Sen Memorial Hospital and the Sun Yat-Sen University, Guangzhou, China. A total of 1860 patients who underwent coronary angiography for presumptive CAD were enrolled from the departments of cardiology at Guangzhou General Hospital of Guangzhou Military Direct, Sun Yat-Sen Memorial Hospital and Zhujiang Hospital in Guangzhou, China, between December 2008 and September 2010. 287 patients with medical illnesses such as acute infection, chronic hepatic dysfunction or nutritional derangements (folate, vitamin B_12_, and vitamin B_6_), malignancies, and other severe medical illnesses were excluded. Of the 1573 patients, 53 patients with insufficient stored blood specimens for tHcy and tCys measurement were excluded. 1015 of the remaining 1520 patients (66.7%) participated in the dietary questionnaire investigation and were included in the present analysis. All patients were free of drugs, such as anticancer agents, which would influence the plasma homocysteine levels. CAD was defined as diameter stenosis ≥ 50% of coronary angiography in one or more major coronary arteries. Hypertension was defined as a systolic pressure of > 140 mmHg and/or diastolic pressure of > 90 mmHg, or patients who were receiving antihypertensive medication. Hyperlipidemia was defined as a plasma total cholesterol level ≥ 200 mg/dL and/or a LDL-cholesterol level ≥ 130 mg/dL or using lipid lowering drugs at the time of investigation. Diabetes mellitus was considered to be present if there was a history of diabetes, a fasting blood glucose level of ≥126 mg/dL, or if the patient was taking antidiabetic medication.

Face-to-face interviews were conducted in addition to a host of health-related and anthropometric data collected in the hospital by trained investigators. Detailed information on age, current smoking status, alcohol consumption, tea use, a family history of CAD (defined as an immediate relative receiving a diagnosis of having CAD before the age of 60 years), a history of hypertension, diabetes, and hyperlipidemia was collected by using a standardized questionnaire during a clinical visit. Habitual dietary intake was assessed by a validated food frequency questionnaire [19], and nutrient consumption was calculated based on the Chinese Food Composition Tables 2002 [20]. Before the data were entered into a database, the questionnaires were checked by a trained interviewer for missing data and completeness. At the same clinical visit, body weight, height, waist and hip circumferences, were measured by using standard techniques in triplicate and the averaged values were used for the analysis. Body mass index was calculated by dividing the patients’ weight in kilograms by their height in squared meters. The study was approved by the Institutional Review Boards of Sun Yat-sen University Health Science Center. All invited participants provided informed written consent.

### Assessment of dietary intake

Usual dietary intake over the preceding 12 months was assessed with an interviewer-administered Chinese food frequency questionnaire developed from 24-h dietary recall data from the 1992 China Health and Nutrition Survey [21]. The major food item contributors to intake from this study were compiled, and some items were deleted or additional items added according to the eating practices common in Guangzhou. The questionnaire lists 130 individual food items. Subjects were asked to recall the frequency of consumption of individual food items (number of times/day, /week, /month or /year), and the estimated portion size, using local weight units [liang (50 g) and jin (500 g)] or natural units (small, medium or large). Questions regarding dietary supplement intake were included and the product names of the most-used supplements in China were used. Dietary intakes were calculated by using a Chinese food-composition database and the frequency of each food item was then converted to per-day consumption [20]. For example, a response of 5 servings/mo was converted to 0.16 servings/d or a response of 1 servings/wk was converted to 0.14 servings/d. The validity and some of the food items of this questionnaire have been previously described for their associations with B vitamins and homocysteine [22].

We assessed daily consumption of the main foods contributing to the intake of total protein, animal protein, and plant protein. The selected food groups contributed to animal protein were including red meats, chicken, milk and dairy products, organ meats, egg, and fish. The selected food groups contributed to the plant protein were including cereals (rice and flour products including wheat and corn based flour), vegetables, fruit and fruit juices, legumes and soybean products, nuts, tea, caffeine, and plant protein drinks; the total protein intakes were calculated by the sum of animal and plant protein.

### Biochemical measurements

Blood was drawn after a 12-h overnight fast. Plasma was separated by centrifugation at 2500 g for 20 min at 4°C. All plasma samples were divided into aliquots and stored at -80°C until analysis. The plasma tHcy and tCys were simultaneously measured by high-performance liquid chromatography (HPLC) with fluorescence detection [23]. Plasma folate was measured by 96-well plate microbiological assay with *Lactobacillus casei* as the assay organism [24]. Plasma vitamin B_12_ was measured using Immulite Chemiluminescent kits according to the manufacturer’s instruction and performed with ARCHITECT 2000 analyzer (Abbott Laboratories). Plasma pyridoxal-5’-phosphate (PLP, the active circulating form of vitamin B_6_) was determined by HPLC with fluorometric detection [25].

Laboratory tests were preformed in the Guangzhou Military General Hospital including serum measurements of HDL, LDL, total cholesterol, triglycerides, glucose, and creatinine.

### Statistical analysis

Continuous variables were expressed as means ± SD and categorical variables as *n* (%). Categorical variables were analyzed by chi-square to compare the frequencies across the quartiles of each dietary protein intake as percentage of total energy intake. For continuous variables, one-way ANOVA with Bonferroni post hoc test or analysis of covariance (ANCOVA) after adjustment for age, sex, BMI, total energy intake, and total fat intake was used to compare the means across the quartiles of each dietary protein. Hyperhomocysteinemia and hypercysteinemia were defined as plasma tHcy concentration > 16 μmol/L and plasma tCys concentration > 300 μmol/L. Logistic regression analysis was used to examine the association between the quartiles of the protein intake as percentage of total energy intake (independent variable) and hyperhomocysteinemia or hypercysteinemia (dependent variable) with and without adjustment for age, sex and total energy intake, total fat intake, BMI, smoking, alcohol use, tea use, hypertension, diabetes, folate, PLP, vitamin B_12_, HDL, LDL, and creatinine. Values are expressed as OR (95% CI).

For the assessment of plasma tHcy and tCys determinants, values for tHcy and tCys were logarithmic transformed as dependent variables, multiple linear regression were performed with adjustment for age, sex, total energy intake, total fat intake, BMI, plasma vitamins status, and lipids profiles in different models. Furthermore, Gaussian generalized additive regression models [26], as implemented in S-PLUS for WINDOWS software (version 8.0; Insightful Corporation, Seattle, WA), were used to generate graphic representations of the dose–response relations between tHcy, tCys concentrations and the intake of different types of protein, after adjustment for age, sex, energy intake, BMI and WHR [10]. On the y-axis, this nonparametric model generates a reference value of zero that approximately corresponds to the tHcy and tCys concentrations associated with the mean intakes of different types of protein for all subjects. For other analyses, we used SPSS for WINDOWS software (release 13.0; SPSS Inc, Chicago, IL). All *P* values are two-sided, and values < 0.05 were considered significant.

## Results

Demographic and anthropometric information of patients according to quartiles of different protein intakes as percentage of total energy intake are presented in Table 1. The patients in highest quartile of plant protein intake were older than those in lowest quartile. The proportion of male was higher in highest quartile of total protein and animal protein intakes and lower in highest quartile of plant protein intake compared with the lowest quartile. Total energy and total fat intakes were higher in the 3rd and 4th quartiles of the total protein intake compared with the lowest quartile. Similar significant trends were observed for cigarette smoking and alcohol consumption in the quartiles of different protein intakes. The proportion of tea use increased across the quartiles of total protein and animal protein intakes and decreased across the quartiles of plant protein intake.

**Table 1:** Characteristics of study population in the lowest to highest quartiles of different dietary protein intake

	**Total protein intake (% of total energy intake)**					**Animal protein intake (% of total energy intake)**					**Plant protein intake (% of total energy intake)**				
**Characteristics**	**Q1 (<10.1%)**	**Q2 (10.1-12.8%)**	**Q3 (12.9-15.9%)**	**Q4 (>15.9%)**	***P***^**a**^	**Q1 (<3.7%)**	**Q2 (3.7-5.6%)**	**Q3 (5.7-8.4%)**	**Q4 (>8.4%)**	***P***^**a**^	**Q1 (<4.9%)**	**Q2 (4.9-6.6%)**	**Q3 (6.7-8.4%)**	**Q4 (>8.4%)**	***P***^**a**^
*n*	254	254	254	253		254	254	254	253		254	254	254	253	
Age, y	61 ± 11	62 ± 12	62 ± 11	61 ± 12	0.48	61 ± 11	62 ± 11	62 ± 11	61 ± 12	0.47	60 ± 12	61 ± 11	62 ± 11	63 ± 11	0.01
Male, n (%)	138(54)	162(63)	164(64)	188(74)	<0.001	139(54)	175(68)	162(63)	176(69)	0.002	195(76)	169(66)	146(57)	142(56)	<0.001
BMI, kg/m^2^	24.7 ± 3.9	24.6 ± 3.9	24.2 ± 3.8	24.3 ± 3.7	0.39	24.6 ± 3.5	24.5 ± 4.0	24.5 ± 4.2	24.2 ± 3.7	0.73	24.9 ± 3.9	24.2 ± 4.0	24.5 ± 4.0	24.3 ± 3.4	0.15
WHR	0.97 ± 0.06	0.93 ± 0.08	1.01 ± 0.07	0.91 ± 0.07	0.27	1.01 ± 0.08	0.97 ± 0.06	0.92 ± 0.06	0.91 ± 0.07	0.23	0.94 ± 0.07	0.92 ± 0.06	0.96 ± 0.09	0.93 ± 0.07	0.28
Total energy intake, MJ/d	8.1 ± 3.4	8.2 ± 4.2	9.2 ± 5.3	11.2 ± 5.8	<0.001	9.3 ± 4.5	9.4 ± 4.8	8.7 ± 4.1	9.0 ± 5.2	0.21	12.0 ± 5.7	9.2 ± 4.4	7.9 ± 3.3	7.5 ± 3.6	0.001
Total fat intake,% of total energy intake	23.5 ± 11.7	30.0 ± 9.7	29.8 ± 8.8	31.1 ± 8.6	<0.001	24.4 ± 11.1	28.2 ± 9.2	30.2 ± 9.4	31.6 ± 9.5	<0.001	26.6 ± 13.5	29.2 ± 9.5	28.9 ± 8.3	29.8 ± 8.2	0.30
Smoker, n (%)	151(59)	117(46)	98(38)	87(34)	<0.001	137(53)	106(41)	116(45)	94(37)	0.001	158(62)	115(45)	92(36)	88(34)	0.001
Alcohol user, n (%)	191(75)	137(54)	109(42)	98(38)	<0.001	74(29)	54(21)	53(20)	39(15)	0.003	87(34)	57(22)	41(16)	35(13)	<0.001
Tea user, n (%)	112(21)	129(24)	136(25)	158(30)	<0.001	117(22)	112(44)	155(61)	151(59)	0.005	197(77)	147(57)	108(42)	83(32)	0.001
Family history of CAD, n (%)	12(4.7)	19(7.5)	8(3.1)	17(6.7)	0.12	9(3.5)	17(6.7)	16(6.3)	14(5.5)	0.41	14(5.5)	11(4.3)	17(6.7)	14(5.5)	0.71
Hypertension, n (%)	167(64)	170(67)	154(60)	147(57)	0.12	165(65)	166(65)	162(63)	142(56)	0.10	155(61)	158(62)	162(63)	160(63)	0.92
Hyperlipidmia, n (%)	27(10)	42(16)	39(15)	34(13)	0.24	33(13)	35(13)	36(14)	38(15)	0.93	31(12)	36(14)	35(13)	40(15)	0.70
Diabetes, n (%)	61(24)	61(24)	56(22)	62(24)	0.90	67(26)	60(23)	59(23)	54(21)	0.61	60(23)	49(19)	64(25)	67(26)	0.24

Values are means ± SD or n (%).

^a^*P*-value was based on ANOVA when row variable was continuous (age, BMI, WHR, total energy intake, and total fat intake) and χ^2^ test when row variable was categorical.

Plasma concentrations of tHcy, tCys and other biomarkers according to quartiles of dietary protein intakes are shown in Table 2. Plasma tHcy concentrations were higher in the highest quartiles of the total and animal protein intakes compared with the lowest quartile. In contrast, plasma tHcy was significantly lower in the highest quartile of the plant protein intake compared with the lowest quartile. Similarly, plasma tCys concentrations were higher in the highest quartile of the total and animal protein intakes compared with the lowest quartile. Circulating tHcy concentration correlated more with those of folate (r = -0.24; *P* < 0.001) and vitamin B_12_ (r = -0.23; *P* < 0.001) than plasma tCys concentration with folate (r = -0.09; *P* = 0.004) and vitamin B_12_ (r = -0.03; *P* = 0.254). However, there was no significant correlation between plasma tHcy or tCys concentrations and PLP levels.

**Table 2:** **Circulating concentrations of biomarkers by quartiles of different dietary protein intake**^**a**^

	**Total protein intake**			**Animal protein intake**			**Plant protein intake**		
**Characteristics**	**Q1**	**Q4**	***P***	**Q1**	**Q4**	***P***	**Q1**	**Q4**	***P***
*n*	254	253		254	253		254	253	
Homocysteine, μmol/L	14.4 ± 6.1	16.7 ± 7.8	<0.001	13.3 ± 5.5	17.9 ± 8.3	<0.001	17.1 ± 7.7	13.5 ± 5.1	<0.001
Cysteine, μmol/L	245 ± 44	252 ± 51	0.01	244 ± 44	249 ± 52	0.06	251 ± 49	247 ± 48	0.54
Folate, nmol/L	9.84 ± 3.27	9.85 ± 3.65	0.96	10.16 ± 3.61	9.38 ± 3.39	0.08	9.22 ± 3.21	10.46 ± 3.85	0.001
PLP, nmol/L	35.7 ± 9.76	36.2 ± 9.87	0.30	36.6 ± 9.4	35.9 ± 9.3	0.23	34.9 ± 9.3	38.5 ± 9.8	0.001
Vitamin B_12_, pmol/L	311 ± 66	307 ± 70	0.58	319 ± 72	302 ± 69	0.20	298 ± 64	326 ± 73	<0.001
Triglycerides, mmol/L	1.87 ± 1.55	1.77 ± 1.08	0.55	1.99 ± 1.61	1.84 ± 1.20	0.17	1.89 ± 1.34	1.79 ± 1.05	0.67
Total cholesterol, mmol/L	4.66 ± 1.25	4.72 ± 0.98	0.73	4.68 ± 1.27	4.73 ± 1.00	0.56	4.62 ± 1.17	4.75 ± 1.07	0.38
LDL cholesterol, mmol/L	2.96 ± 1.08	2.94 ± 0.90	0.91	2.97 ± 1.08	2.89 ± 0.89	0.46	2.92 ± 1.04	3.04 ± 0.92	0.53
HDL cholesterol, mmol/L	1.06 ± 0.29	1.12 ± 0.31	0.10	1.08 ± 0.30	1.12 ± 0.31	0.12	1.04 ± 0.29	1.10 ± 0.28	0.15
Fasting plasma glucose, mmol/L	6.50 ± 3.34	6.34 ± 2.55	0.76	6.54 ± 3.20	6.47 ± 2.64	0.54	6.61 ± 3.03	6.52 ± 2.77	0.41
Creatinine, μmol/L	90.5 ± 53.8	81.4 ± 28.6	0.01	90.4 ± 56.0	81.1 ± 29.2	0.11	93.6 ± 57.4	83.0 ± 35.7	0.03

^a^Values are mean ± SD. The means were evaluated by ANCOVA with adjustment for age, sex, BMI, total energy intake, and total fat intake.

Further, we explored the association between the different diet protein intakes and hyperhomocysteinemia or hypercysteinemia (Table 3). After adjustment for age, sex, total energy intake, total fat intake, BMI, smoking, alcohol use, tea use, hypertension, diabetes, folate, PLP, vitamin B_12_, HDL, LDL, and creatinine, the highest quartile of the total protein intake was positively associated with hyperhomocysteinemia (OR: 2.63, 95% CI: 1.70-4.07) and hypercysteinemia (OR: 1.69, 95% CI: 1.02-2.87). Similarly, the highest quartile of animal protein intake was positively associated with hyperhomocysteinemia. Compared with the lowest quartile of intake, the full-adjusted OR was 4.14 (95% CI: 2.67-6.43) for the 4th quartile. There was an inverse relationship between the quartiles of plant protein intake and hyperhomocysteinemia but not hypercysteinemia. Compared with the reference group, reduced odds for hyperhomocysteinemia with the plant protein intake was 24% (95% CI: 0.51-1.13) for the 2nd quartile, 37% (95% CI: 0.41-0.96) for the 3rd quartile, and 41% (95% CI: 0.38-0.91) for the 4th quartile when the model was adjusted for all above confounders.

**Table 3:** OR and 95% CI of hyperhomocysteinemia and hypercysteinemia by quartiles of different dietary protein intake

	**Hyperhomocysteinemia**^**a**^				**Hypercysteinemia**^**a**^			
	**Q1**	**Q2**	**Q3**	**Q4**	**Q1**	**Q2**	**Q3**	**Q4**
*n*	253	254	254	254	253	254	254	254
Total protein intake								
Crude OR (95% CI)^b^	1	1.20(0.84-1.71)	1.30(0.91-1.85)	1.94(1.36-2.77)^**^	1	1.20(0.70-2.06)	1.60(0.96-2.68)	1.78(1.07-2.96)^*^
Adjusted OR (95% CI)^c^	1	1.30(0.89-1.91)	1.37(0.94-1.99)	2.13(1.45-3.12)^**^	1	1.16(0.66-2.04)	1.67(0.97-2.86)	1.73(1.03-2.92)^*^
Adjusted OR (95% CI)^d^	1	1.48(0.98-2.25)	1.57(1.02-2.41)^*^	2.63(1.70-4.07)^**^	1	1.14(0.64-2.03)	1.56(0.89-2.75)	1.69(1.02-2.87)^*^
Animal protein intake								
Crude OR (95% CI)^b^	1	1.46(1.01-2.10)^*^	1.79(1.25-2.58)^*^	3.32(2.30-4.79)^**^	1	1.27(0.76-2.13)	1.34(0.80-2.24)	1.50(0.91-2.49)
Adjusted OR (95% CI)^c^	1	1.51(1.03-2.22)^*^	1.87(1.27-2.75)^*^	3.73(2.52-5.54)^**^	1	1.28(0.74-2.21)	1.45(0.84-2.51)	1.59(0.93-2.70)
Adjusted OR (95% CI)^d^	1	1.56(1.03-2.38)^*^	2.01(1.31-3.08)^*^	4.14(2.67-6.43)^**^	1	1.34(0.77-2.36)	1.51(0.85-2.68)	1.60(0.91-2.80)
Plant protein intake								
Crude OR (95% CI)^b^	1	0.77(0.54-1.10)	0.51(0.35-0.72)^**^	0.46(0.32-0.66) ^**^	1	1.01(0.62-1.62)	0.91(0.55-1.48)	0.88(0.53-1.44)
Adjusted OR (95% CI)^c^	1	0.77(0.53-1.10)	0.54(0.37-0.79)^*^	0.47(0.32-0.68)^**^	1	1.11(0.66-1.86)	1.02(0.61-1.70)	0.90(0.53-1.51)
Adjusted OR (95% CI)^d^	1	0.76(0.51-1.13)	0.63(0.41-0.96)^*^	0.59(0.38-0.91)^*^	1	1.28(0.73-2.26)	1.12(0.64-1.93)	0.96(0.56-1.64)

^a^Cut-off points are > 16 μmol/L for plasma homocysteine and > 300 μmol/L for plasma cysteine.

^b^Values are OR (95% CI) from logistic regression.

^c^Values are OR (95% CI) from logistic regression adjusted for age, sex, total energy intake, total fat intake, and BMI.

^d^Values are OR (95% CI) from logistic regression adjusted for age, sex, total energy intake, total fat intake, BMI, smoking, alcohol use, tea use, hypertension, diabetes, folate, PLP, vitamin B12, HDL, LDL, and creatinine. Asterisks indicate difference from lowest quartiles of different dietary protein intake. ^*^P < 0.05, ^**^P < 0.001.

**Figure 1:**
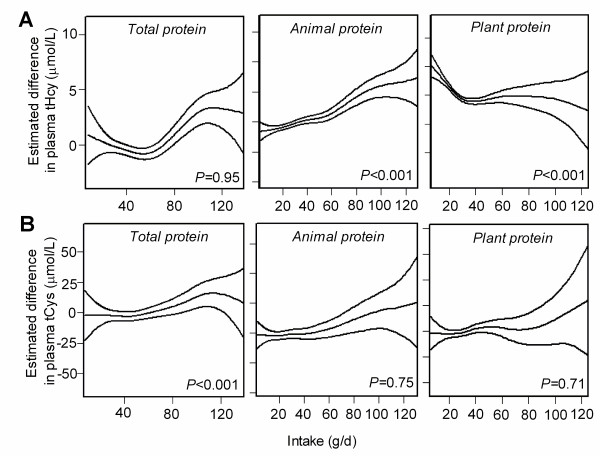
**Dose–response relationship between dietary protein and plasma tHcy, tCys concentrations.** Estimated mean (and 95% CI) plasma tHcy **(A)** and tCys **(B)** concentrations according to the intake of different types of protein after adjustment for age, sex, body mass index, waist hip ratio and total energy intake by additive Gaussian generalized regression model. *n* = 965. Middle lines, the estimated dose–response curves; up and below lines, 95% CIs. *P* values are from corresponding multiple linear regression analyses. The lowest and highest 2.5 percentiles of protein intakes are not included.

To examine the relationship between different protein intakes and plasma tHcy and tCys concentrations, the dose–response curves between tHcy (Figure 1A) and tCys (Figure 1B) and the types of protein intakes in all subjects are shown. We found a positive trend between tHcy and animal protein intake, and an inverse trend between tHcy and plant protein intake, and a positive trend between tCys and total protein intake. We also repeated the regression analysis with the intakes of different types of protein as independent variables. Intakes of total and animal protein were positively associated with plasma tHcy (β = 0.20, *P* < 0.001; β = 0.35, *P* < 0.001, respectively), the plant protein intake was inversely associated with plasma tHcy (β = -0.19, *P* < 0.001). Furthermore, the intakes of total protein and animal protein were also significant predictors of tCys (β = 0.13, *P* = 0.001; β = 0.10, *P* = 0.001, respectively). These significant associations remained after adjustment for vitamins status, lipids profiles, and other covariates in different models (Table 4).

**Table 4:** **Multivariate linear regression models for predicting the determinants of the plasma homocysteine and cysteine levels**^**a**^

	**Homocysteine (μmol/L)**						**Cysteine (μmol/L)**					
	**Model 1**		**Model 2**		**Model 3**		**Model 1**		**Model 2**		**Model 3**	
**Characteristics**	**β**^**b**^	***P***^**c**^	**β**^**b**^	***P***^**c**^	**β**^**b**^	***P***^**c**^	**β**^**b**^	***P***^**c**^	**β**^**b**^	***P***^**c**^	**β**^**b**^	***P***^**c**^
Age (y)	0.06	0.03	0.02	0.47	0.03	0.30	0.01	0.88	0.01	0.91	0.01	0.90
Sex	0.03	0.29	0.05	0.08	0.05	0.12	0.01	0.75	0.01	0.75	0.02	0.52
BMI, kg/m^2^	0.05	0.08	0.04	0.16	0.05	0.09	0.01	0.79	0.01	0.88	0.01	0.89
Total energy intake, MJ/d	0.03	0.23	0.02	0.34	0.03	0.25	0.02	0.53	-0.01	0.81	0.01	0.84
Total fat intake^d^	0.02	0.48	0.02	0.60	-0.01	0.63	-0.03	0.35	-0.03	0.39	-0.02	0.53
Total protein intake^d^	0.20	**<0.001**	0.39	**<0.001**	0.40	**<0.001**	0.13	**0.001**	0.12	**0.002**	0.12	**0.002**
Animal protein intake^d^	0.35	**<0.001**	0.31	**<0.001**	0.33	**<0.001**	0.10	**0.001**	0.09	**0.003**	0.10	**0.003**
Plant protein intake^d^	-0.19	**<0.001**	-0.28	**<0.001**	-0.35	**<0.001**	-0.03	0.37	-0.02	0.68	-0.01	0.76
Folate, nmol/L			-0.17	**<0.001**	-0.16	**<0.001**			-0.09	**0.006**	-0.09	**0.007**
PLP, nmol/L			0.03	0.37	0.03	0.24			-0.05	0.10	-0.05	0.09
Vitamin B_12_, pmol/L			-0.19	**<0.001**	-0.15	**<0.001**			0.05	0.08	0.05	0.13
Triglycerides, mmol/L					0.01	0.93					0.04	0.30
Total cholesterol, mmol/L					-0.03	0.59					-0.05	0.11
LDL cholesterol, mmol/L					0.07	0.17					0.01	0.80
HDL cholesterol, mmol/L					-0.01	0.91					-0.02	0.63
Fasting plasma glucose, mmol/L					0.01	0.94					-0.05	0.11
Creatinine, μmol/L					0.12	**<0.001**					0.03	0.30

^a^All variables in each model were entered simultaneously.

^b^β values are standardized coefficients.

^c^Bold *P* values are statistically significant.

^d^% of total energy intake.

## Discussion

In the present study, using dietary data from validated FFQs, we observed significant positive association between dietary animal-protein intake and plasma tHcy concentrations, and inverse relationship of plant-protein intake and plasma tHcy concentrations in coronary angiographic subjects. Furthermore, we found significant positive association between total-protein intake and plasma tCys concentrations. These associations between dietary protein and plasma tHcy or tCys concentrations appeared to be independent of age, sex, and other potential confounding factors.

The positive association between dietary intake of animal-protein and plasma tHcy has also been observed in a population-based study in Pakistani [27]. The association was strong and statistically significant in the population of the present study. This may be due to an increased intake of red meats, chicken, milk and dairy products, organ meats, and egg (rich sources of methionine) [28]. The only dietary precursor of homocysteine is the essential amino acid methionine [7]. Furthermore, in Chinese diets, meat and chicken are main sources of saturated fat. It has been shown that an increased dietary intake of saturated fat caused an increase in plasma tHcy concentrations [10,29]. Moreover, fish and fish oil supplements are rich sources of very-long-chain n-3 fatty acids. Dietary supplementation studies have reported conflicting results with respect to the effect of very-long-chain n-3 fatty acids on plasma tHcy concentrations. Some studies showed supplementation with fish or fish oil did not affect tHcy concentrations in hyperlipidemic subjects but increased tHcy concentrations in normlipidemic subjects [30-32]. On the other hand, some studies reported fish oil could reduce the tHcy concentrations in hyperlipidemic and CVD patients [33,34]. Although we did not evaluate separately the association between the intake of fish and plasma tHcy concentrations, our present study considered the fish as sources of dietary animal-protein and showed a positive association between the dietary intake of animal-protein and plasma tHcy concentrations. Hence, the dietary intake of animal protein is an important determinant of plasma tHcy concentrations.

The inverse association between high plant-protein intake and plasma tHcy concentrations in present study is similar to the findings of some other studies [22,28]. It is well known that the high plant-protein diet patterns include cereals (rice and flour products including wheat and corn based flour), vegetables, fruit and fruit juices, legumes and soybean products, which are important food sources of folate and B vitamins. In the transmethylation pathway, homocysteine is remethylated to methionine in the presence of the enzyme methionine synthase. Folate and vitamin B_12_ are cofactors for methionine synthase, and therefore, are necessary for removal of homocysteine by transmethylation. Results from the Framingham Heart Study and the Health Professionals Follow-up Study all showed that frequent consumption of fruit and vegetables was associated with high plasma folate and low tHcy concentrations [28,35]. Furthermore, in the DASH study, after consuming a combination diet rich in fruit, vegetables and low fat diary products and reduced in saturated and total fat for 8 weeks, the plasma tHcy concentration was decreased and the change differed significantly from the control diet [36]. In the present study, we observed, as have others, that plasma tHcy concentration was inversely associated with plasma folate or vitamin B_12_ concentrations. Hence, the positive relationship of increased intake of the high plant-protein diet with plasma folate and vitamin B_12_ may partly explain why we found the low levels of plasma tHcy in individuals in the top quartile of high plant-protein dietary intake (Figure 1A), indicating that adequate consumption of fresh fruits and vegetables could be beneficial in reducing the risk of hyperhomocysteinemia. It has been shown that dietary intake is an important contributor to plasma vitamin B_12_[37]. In the present study, we found plasma vitamin B_12_ was associated with plant protein intake but not with animal protein intake. This may be due to that plant protein coming from fermentation soybean products and organic plant products such as laver slice and mushrooms, which are frequently consumed by Chinese populations, contains enriched vitamin B_12_. Although vitamin B_12_ levels were lower in lowest quartile of the plant protein intake than that in lowest quartile of animal protein intake, this suggests that animal products are better sources of vitamin B_12_ than plant products, particularly milk and fish. Because vitamin B_12_ in meat is less bioavailable than that in milk and fish [37], and we did not analyze the detailed associations between meat or milk and fish and vitamin B_12_, hence, combined different animal-source proteins may result in no significant association between vitamin B_12_ and animal protein intake.

Furthermore, we observed an adverse effect of high consumption of total protein on hypercysteinemia (Table 3). In multivariate regression analyses, we also found significant dose–response associations between total protein and animal protein intakes and plasma tCys concentrations. In addition, previous studies showed that plasma tCys was strongly related to several cardiovascular risk factors such as high cholesterol [5,11]. But few studies reported the effect of different dietary proteins on plasma tCys concentrations. In the present study, the high intake of total protein was positively associated with plasma tCys concentrations. This may be due to the high intake of total protein including high animal protein dietary products which are rich sources of methionine, the dietary precursor of homocysteine. Homocysteine is then degraded to cysteine by the sequential actions of two vitamin B_6_-dependent enzymes. In view of the strong association between tCys and total protein intake, it would be important to test whether tCys is associated with total plasma protein or plasma albumin, and is thus a marker of protein availability or nitrogen balance. Further studies are needed to demonstrate this hypothesis in the future.

An advantage of our study is that we conducted the study in a large population-based sample, using a validated FFQ. This allowed the investigation of the relationship of dietary protein intakes and tHcy and tCys concentrations. A few limitations should be considered when interpreting the findings of the present study. Collection of dietary data using a FFQ has inherent potential problems related to inaccuracy and potential misclassification in the estimation of protein intakes. The recall of past diets raises concerns regarding the possibility of recall bias among patients if their diets changed as a result of their diagnosis. It is also possible that the dietary intake interview reflected changes due to medical advice. To minimize the recall bias, patients were constantly reminded to report on their dietary intake before diagnosis with CAD. Furthermore, although we found plasma folate and vitamin B_12_ levels were significantly related to tHcy and adjusted for them in multivariate models, there are few patients with using B-vitamin supplements in our study population, this does not negate an effect of dietary vitamin supplements on plasma tHcy and tCys concentrations, which may mediate the relationship between dietary protein intake and tHcy and tCys concentrations. Finally, it was revealed that distinct dietary patterns were influenced by socio-economic and lifestyle characteristics. China has achieved remarkable economic progress in recent years. Accompanied with these rapid economic changes, dietary pattern is shifting from the traditional pattern with high intake of cereals and vegetables and low intake of animal foods to the Western pattern with high intake of animal foods and other high-energy-dense foods. Hence, there may be some differences or similarities of dietary protein levels and sources between Chinese and Western diets. For example, the levels of total dietary protein intake in Chinese populations may be lower than those in Western populations. The sources of dietary protein in Chinese diets are mainly from plant protein such as cereals, legumes and soybean products, whereas the sources of dietary protein in Western diets are mainly from animal protein such as red meats, chicken, milk and dairy products. These different sources of dietary protein between Chinese and Western diets may explain the differences of dietary protein levels. Consequently, it should be cautious to extrapolate our findings to other Western populations.

## Conclusion

In conclusion, increased intake of a high animal-protein diet increased the risk of hyperhomocysteinemia. On the contrary, the high plant-protein diet showed a protective effect toward the development of hyperhomocysteinemia. Furthermore, we observed that the increased intake of total protein in this population could, perhaps, be one of the reasons for hypercysteinmia. Hence, the dietary pattern that moderate decrease in the total protein intake and changing the categories of protein by increasing consumption of a high plant-protein diet and reducing intake of a high animal-protein diet should be recommended to keep the concentrations of plasma tHcy and tCys within acceptable limits.

## Abbreviations

BMI: Body mass index; CAD: Coronary artery disease; CVD: Cardiovascular disease; FFQ: Food frequency questionnaire; HDL: High density lipoprotein; HPLC: High-performance liquid chromatography; LDL: Low density lipoprotein; PLP: Pyridoxal-5’-phosphate; tCys: Total cysteine; tHcy: Total homocysteine; WHR: Waist hip ratio.

## Competing interests

The authors declare that they have no competing interests.

## Authors’ contributions

The authors responsibilities are as follows-: WL, as the principal investigator, was responsible for the concept and design of the study. YZ, MW, and XL conducted data analysis; YX and WL drafted the manuscript and had primary responsibility for final content; YX and MX provided statistical expertise, extensively reviewed, and edited the manuscript; and all authors were involved in interpretation of results and revision of the manuscript and approved the final version of the manuscript.
